# Breath Stacking: Acute Effects on Cough Peak Flow and Chest Wall Volumes of Healthy Subjects

**DOI:** 10.3390/jfmk10040421

**Published:** 2025-10-29

**Authors:** Ana Cristina de Medeiros Garcia Maciel, Vanessa Regiane Resqueti, Jéssica Danielle Medeiros da Fonseca, Illia Nadinne Dantas Florentino Lima, Matías Otto-Yáñez, Rêncio Bento Florêncio, Andrea Aliverti, Guilherme Augusto de Freitas Fregonezi, Arméle de Fátima Dornelas de Andrade

**Affiliations:** 1 PneumoCardioVascular Lab/HUOL, Hospital Universitário Onofre Lopes, Departamento de Fisioterapia, Universidade Federal do Rio Grande do Norte, Natal 59078-970, RN, Brazil; anacristinamgmaciel@gmail.com (A.C.d.M.G.M.); vanessa.resqueti@ufrn.br (V.R.R.); jessy_nielle@hotmail.com (J.D.M.d.F.); illialima@yahoo.com.br (I.N.D.F.L.); 2 Laboratório de Inovação Tecnológica em Reabilitação, Departamento de Fisioterapia, Universidade Federal do Rio Grande do Norte, Natal 59078-970, RN, Brazil; 3 Grupo de Investigación en Salud, Funcionalidad y Actividad Física (GISFAF), Kinesiología, Facultad de Ciencias de la Salud, Universidad Autónoma de Chile, Santiago 8320000, Chile; matiasotto.kine@gmail.com; 4 Instituto de Patologia Tropical e Saúde Pública, Departamento de Saúde Funcional, Universidade Federal de Goiás (UFG), Goiás 74690-900, GO, Brazil; rencio_bento@ufg.br; 5 Dipartimento di Elettronica, Informazione e Bioingegneria, Politecnico di Milano, 20156 Milan, Italy; andrea.aliverti@polimi.it; 6 Departamento de Fisioterapia, Universidade Federal de Pernambuco, Recife 55265-000, PE, Brazil; armeledornelas@yahoo.com

**Keywords:** respiratory physiotherapy, breath stacking, lung volumes, cough peak flow, body posture

## Abstract

**Objectives:** The aim of this study was to investigate the acute physiological effects of the BS on CPF and chest wall volumes in healthy individuals, comparing two body positions: supine and 45° trunk inclination. **Methods:** Observational, analytical, and cross-sectional study conducted with 14 healthy individuals (7 males) who underwent the BS in two different body positions. CPF, tidal chest wall volumes and the contribution of thoracoabdominal compartments were assessed using Optoelectronic Plethysmography. Statistical analyses included two-way ANOVA and Bonferroni post hoc tests, with a significance level of 5%. **Results:** A significant increase in CPF was observed after the BS in the inclined position (*p* < 0.05), with no relevant changes in the supine position. Tidal chest wall volumes also increased in both positions, with a more pronounced effect in the 45° inclination (*p* < 0.05). The volume increase was predominantly thoracic, with a ventilatory redistribution favouring the pulmonary rib cage compartment, especially in the inclined posture. **Conclusions:** The BS produced immediate beneficial physiological effects in healthy individuals, with greater efficacy in the inclined position, enhancing expiratory flow and thoracic ventilation. These findings support the clinical applicability of BS as a physiotherapeutic strategy even in populations without respiratory dysfunction.

## 1. Introduction

A reduction in lung volume is a common feature across various clinical conditions, resulting in a restrictive respiratory pattern. Such impairment is frequently observed in patients undergoing thoracic, cardiac, and abdominal surgeries, as well as in individuals with cystic fibrosis, obesity, and neuromuscular diseases. These alterations may lead to respiratory complications and negatively impact patients’ quality of life [1,2].

The decrease in operational lung volumes reduces the elastic recoil of the lungs and increases airway resistance—factors that contribute to a decline in cough peak flow (CPF), a key parameter for the effectiveness of mucociliary clearance [3].

Respiratory physiotherapy plays a crucial role in pulmonary re-expansion and in the prevention of pulmonary complications, employing techniques such as Breath Stacking (BS). This technique consists of multiple consecutive inspirations without intermediate expirations, facilitated by a unidirectional valve connected to a facial mask. This mechanism promotes air accumulation in the lungs, stimulates collateral ventilation, and contributes to alveolar recruitment [4].

During the application of the technique, occlusion of the expiratory branch triggers compensatory mechanisms that progressively elevate lung volume until the limit imposed by thoracic compliance and pulmonary elastic recoil is reached. Despite the mechanical disadvantage imposed on the respiratory muscles due to the inspiratory block created by the thoracic cage, continuous insufflation is sustained until inspiratory efforts become insufficient [5].

Although the benefits of BS are well established in clinical populations, the acute physiological effects of this maneuver in healthy individuals remain incompletely understood. Considering that BS enables inspiratory volumes beyond the voluntary inspiratory capacity, it is plausible to assume that it may induce changes in chest wall volumes and enhance CPF even in individuals without respiratory dysfunctions.

Thus, the main objective of the study was to evaluate the immediate effects of BS on cough peak flow of healthy subjects in two different positions: supine and 45° trunk inclination. Second, variations in operational volumes, distribution of these volumes in the chest wall compartments (pulmonary rib cage, abdominal rib cage, and abdominal), and breathing pattern immediately before and after the application of BS were also evaluated.

## 2. Materials and Methods

### 2.1. Study Type

The research consisted of a cross-sectional study with healthy individuals from both sexes and was carried out in the Laboratory of PneumoCardioVascular Physiotherapy and Respiratory Muscles of the Federal University of Rio Grande do Norte within the confines of the World Medical Association Declaration of Helsinki for medical research using human participants and approved by the Research Ethics Committee (approval 163.520/2012). All individuals involved in the study signed a clear and informed consent form (Supplementary Material S1).

### 2.2. Procedures and Data Collection

We included in the analysis 14 subjects (7 males and 7 females; mean age 23.79 ± 2.48 y, weight 67.62 ± 7.09 kg; height 1.69 ± 0.04 m; body mass index of 23.31 ± 1.84 kg/m^2^, FVC of 4.26 ± 0.60 L, and FEV_1_/FVC of 0.83 ± 0.01), who self-reported as healthy with no history of smoking, heart, or lung disease were included in the study. Those who had spirometric values below predicted values (<80% of FVC and FEV_1_), did not adapt, or failed to perform the BS technique were excluded.

The data from lung and respiratory muscle function tests (spirometry, manovacuometry and SNIP), cough peak flows and chest wall volumes were analyzed.

### 2.3. Spirometry

A Koko DigiDoser spirometer (nSpire Health, Longmont, CO, USA) was used to perform pulmonary function test. Three technically acceptable and reproducible forced expiratory curves were obtained for each participant. Variability between them was <5%, and only the curve with the best performance was considered for analysis. FVC, FEV_1_, (forced expiratory volume on the first second) and FEV_1_/FVC in their absolute and relative values were considered for analysis.

Technical procedures, acceptability criteria, reproducibility, and standardization of the different pieces of equipment followed the recommendations of the American Thoracic Society/European Respiratory Society [6]. The predicted reference values for the studied population were calculated according to Pereira et al. [7].

### 2.4. Respiratory Muscle Strength

Maximum inspiratory and expiratory pressures (MIP and MEP) and SNIP were measured using a digital manometer (NEPEB-Labcare, Belo Horizonte, Brazil) with the subjects seated on a chair. MIP was measured starting from residual volume and MEP from total lung capacity, while SNIP was performed starting from functional residual capacity (FRC). Assessments were performed according to ERS recommendations [6]. Data obtained were compared with previous reference values [8], and the highest value of each test was considered for analysis.

### 2.5. Assessment of Cough Peak Flow and Chest Wall Volumes

The optoelectronic plethysmography (OEP) equipment (BTS Bioengineering, Garbagnate Milanese, Italy) was utilized to assess chest wall volume (V_T(CW)_) and the volumes of its compartments. Prior to data collection, the equipment was calibrated to recognize the markers at a frequency of 60 frames per second. In this experiment, the subject was positioned at supine position and a 45° body inclination. At this angle, 52 retro-reflective markers were placed at specific points on the thorax and abdomen. Six photosensitive cameras were positioned around the subject (three on the left and three on the right) to capture movement changes in the markers. This was carried out according to the protocol described by Aliverti et al. [9]. Volumes were obtained following an experimental model according to the Gauss theorem [10,11].

From optoelectronic plethysmography data, the following variables were considered for further analysis: cough peak flow (derived by the volume displaced by chest wall and time during the cough [ΔV_T(CW)_/Δt] [12,13], chest wall tidal volume and its compartments, breathing frequency, end-inspiratory volume, end-expiratory volume, and percentage of contribution of the compartments to V_CW_.

### 2.6. Breath Stacking

The Breath Stacking (BS) technique is a method of breathing that involves stacking one breath upon another, with each successive breath being held for a longer duration than the previous one.

The BS was applied using a silicone mask that covered the nose and mouth, and was equipped with unidirectional valves in the inspiratory and expiratory branches. The inspiratory branch was connected to an analogue ventilometer (Wright MARK 8, nSpire Health GmbH, Germany), while the expiratory branch was occluded. The volunteers were instructed to perform successive inspirations, commencing from FRC, until maximum lung capacity was attained, for a period of approximately 20 s. During this moment, the subjects were instructed to not exhale, holding the amount of air in their lungs The maneuver was terminated when the volunteer signalled discomfort or when there was no increase in inspired volume, as monitored by the ventilometer [14].

### 2.7. Study Design

**Figure 1:**
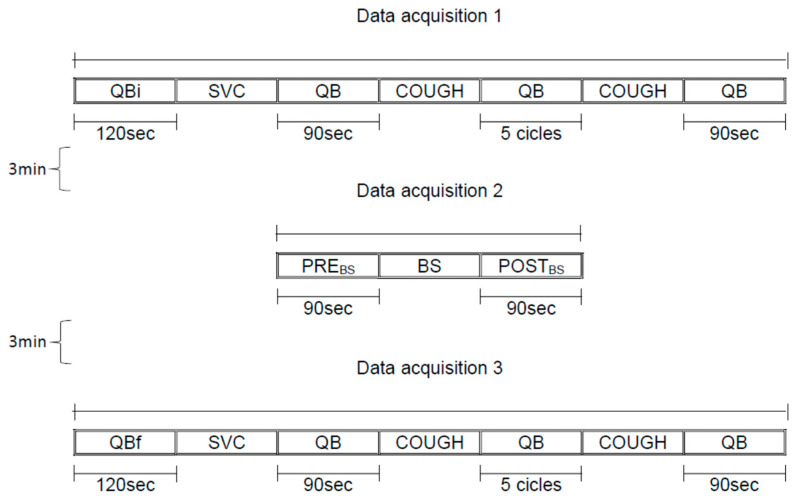
Schematic diagram of the study protocol. Three data acquisitions were made with an interval of 3 min in between: Data acquisition 1: (a) 120 s of quiet breathing initial (QBi), (b) slow vital capacity maneuver (SVC), (c) 90 s of quiet breathing (QB), (d) spontaneous cough, (e) 5 cycles of quiet breathing (QB), (f) spontaneous cough, (g) 90 s of quiet breathing (QB); Data acquisition 2: (a) 90 s of quiet breathing pre Breath Stacking maneuver (PRE_BS_), (b) Breath Stacking (BS), (c) 90 s of quiet breathing post Breath Stacking maneuver (POST_BS_); Data acquisition 3: (a) 120 s of quiet breathing final (QBf), (b) slow vital capacity maneuver (SVC), (c) 90 s of quiet breathing (QB), (d) spontaneous cough, (e) 5 cycles of quiet breathing (QB), (f) spontaneous cough, (g) 90 s of quiet breathing (QB). Duration of data acquisitions: (1) 5 min; (2) 3 min; (3) 5 min.

For each subject, all measurements were made in a single day. After collection of anthropometric data (weight, height, and body mass index), lung function, and respiratory muscle strength, the subjects were positioned with OEP on a standard bed at supine and 45° trunk inclination after 30 min between the positions, and the data were recorded in 3 acquisitions (Figure 1):

(a) Data acquisition 1: 120 s of quiet breathing initial (QBi), slow vital capacity maneuver (SVC), 90 s of quiet breathing (QB), spontaneous cough, 5 cycles of quiet breathing (QB), spontaneous cough, 90 s of quiet breathing (QB);
(b) Data acquisition 2: 90 s of quiet breathing pre Breath Stacking maneuver (PRE_BS_), Breath Stacking (BS), 90 s of quiet breathing post Breath Stacking maneuver (POST_BS_);
(c) Data acquisition 3: 120 s of quiet breathing initial (QBi), slow vital capacity maneuver (SVC), 90 s of quiet breathing (QB), spontaneous cough, 5 cycles of quiet breathing (QB), spontaneous cough, 90 s of quiet breathing (QB).

### 2.8. Statistical Analysis

Statistical analysis was carried out using GraphPad Prism software version 5.0 (GraphPad Software Inc., San Diego, CA, USA). The normality of the data was checked using the Shapiro–Wilk test. The unpaired Student’s *t*-test was used to compare the means between the groups and the two-way ANOVA was used to analyze the variables at the different times (PRE_BS_, BS and POST_BS_), followed by the Bonferroni post hoc test when appropriate. The significance level adopted was 5% (*p* < 0.05).

The power (1 − β) and effect size (ES) were estimated and are detailed in the results section of this study and were calculated using GPower software version 3.1.9.2 (University of Düsseldorf, Kiel, Germany).

## 3. Results

Sixteen subjects were evaluated, but only 14 participated in the study (7 males and 7 females; mean age 23.79 ± 2.48 y, body mass index of 23.31 ± 1.84 kg/m^2^, FVC of 4.26 ± 0.60 L, and FEV_1_/FVC of 0.83 ± 0.01). Two subject was excluded for presenting spirometric values below predicted. The anthropometric characteristics, absolute values, and percentages of the predicted values for lung function and respiratory muscle strength of all subjects are presented in Table 1. The sex-based analysis is described in Table 2.

### 3.1. Effects of Breath Stacking on Cough Peak Flow

**Figure 2:**
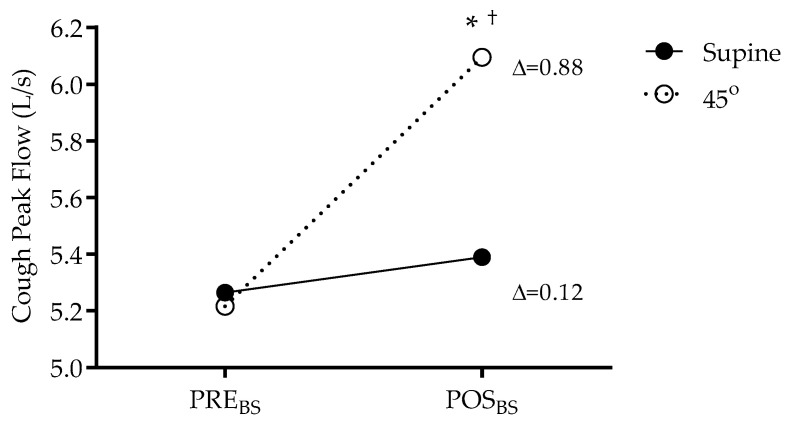
Variation in cough peak flow (CPF) before Breath Stacking (PRE_BS_) and after Breath Stacking (POST_BS_) in the studied sample. *p* < 0.05—Supine vs. 45°—Two-way ANOVA (PRE_BS_ and POST_BS_). ‘*’ indicates intergroup differences, and ‘†’ indicates intragroup differences.

A significant increase in peak cough flow (5.21 vs. 6.09 L/s; *p* < 0.05; 1 − β = 0.99; EZ = 1.25) was observed after the BS in the 45° trunk inclination, as shown in Figure 2.

### 3.2. Effects of Breath Stacking on Total and Operational Chest Wall Volumes

**Figure 3:**
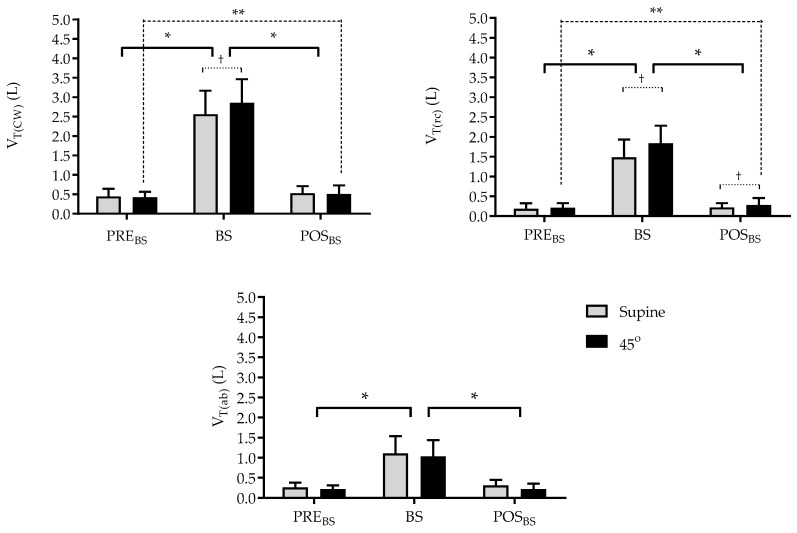
Tidal volume of the chest wall (V_T(cw)_) and its compartments—rib cage (V_T(rc)_) and abdomen (V_T(ab)_)—measured immediately before Breath Stacking (PRE_BS_), during Breath Stacking (BS), and immediately after Breath Stacking (POST_BS_) in the studied sample. * *p* < 0.05—Supine vs. 45°—Two-way ANOVA, across time points (PRE_BS_, BS, and POST_BS_); ** *p* < 0.05—Supine vs. 45°—Two-way ANOVA, between groups; † *p* < 0.05—Interaction between 45° and time points (PRE_BS_, BS, and POST_BS_); Bonferroni post hoc analysis applied across time points and groups.

As illustrated in Figure 3, the chest wall tidal volume (V_T(CW)_) demonstrates variability in response to the BS. The application of the BS resulted in an increase in tidal volume in both positions (Supine position = *p* < 0.0001; 1 − β < 0.99; EZ = 2.66; 45° position = *p* < 0.0001; 1 − β < 0.99; EZ = 3.1). A comparison of the positions during the technique, in the 45° trunk inclination, revealed an increase of 11.2% compared to the supine position (*p* < 0.05). With regard to the POST_BS_ moment, it was observed that the supine position alone continued to demonstrate an increase in V_T(CW)_ (*p* < 0.05). With regard to the distribution of these volumes in the respective compartments, there was an increase in volumes of the pulmonary rib cage compartment (V_T(rcp)_) in POST_BS_ in the 45° trunk inclination (0.211 L vs. 0.279 L; *p* < 0.05), with no significant changes in the abdominal rib cage (V_T(rca)_).

### 3.3. Effects of Breath Stacking on the Contribution of the Compartments to Tidal Volume

The BS influenced the respiratory pattern with different intensities between the supine and 45° trunk inclination. The abdominal compartment was responsible for the greatest contribution to tidal volume at both the PRE_BS_ and POST_BS_ moments in the supine position (61.5% and 59.4%) and in the trunk inclination position (52% and 48.2%), respectively (*p* = 0.02).

**Figure 4:**
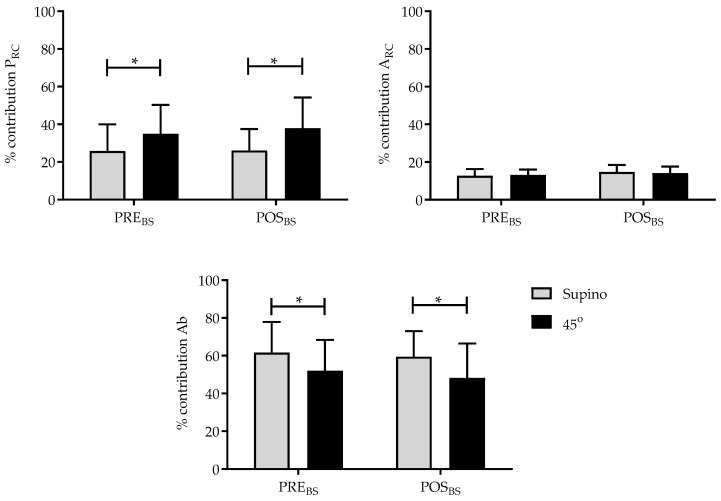
Percentage contribution of the pulmonary rib cage (P_RC_), abdominal rib cage (A_RC_), and abdominal (Ab) compartments immediately before Breath Stacking (PRE_BS_) and immediately after Breath Stacking (POSTBS) in the studied sample. * *p* < 0.05—Supine vs. 45°—Two-way ANOVA (PRE_BS_ and POST_BS_).

The pulmonary rib cage compartment behaved similarly in both positions at PRE_BS_ and POST_BS_, making a greater contribution to tidal volume, with 25.8% at PRE_BS_ in the supine position and 34.9% in the trunk inclination, and 26.0% at POST_BS_ in the supine position and 37.85% in the trunk inclination (*p* = 0.01). There was no difference in the contribution to tidal volume in relation to the abdominal rib cage compartment (Figure 4).

**Figure 5:**
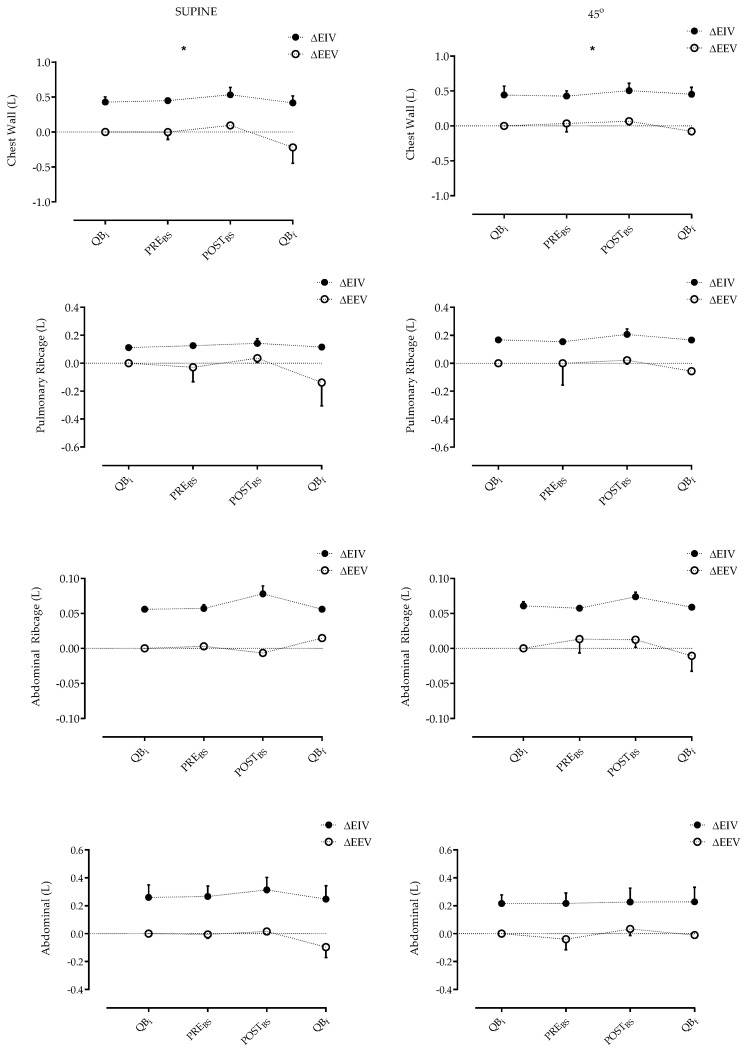
Operational volume of the chest wall and its compartments in the supine and inclined positions during the study protocol in the analyzed sample. (Quiet Breathing Initial [QBi], pre Breath Stacking maneuver [PRE_BS_], post Breath Stacking maneuver [POST_BS_], Quiet Breathing Final [QBf], End-Inspiratory Volume [EIV], End-Expiratory Volume [EEV]). * *p* < 0.05—Supine vs. 45°—Two-way ANOVA (PRE_BS_ and POST_BS_).

With regard to end-inspiratory volume (EIV), there was a significant (*p* < 0.05) increase in volume immediately before BS to immediately after BS in both positions for the chest wall (Δ0.178 L vs. Δ0.145 L) but no significant difference between the positions. There was no difference between the abdominal compartments, which suggests that the volume gain was due to inflation of the rib cage (Figure 5).

## 4. Discussion

The main findings of the study were that BS in healthy individuals was able to (1) increase cough peak flow immediately after the maneuver in the trunk inclination; (2) increase the tidal volume of the chest wall in both positions and (3) modify the breathing pattern.

The selection of the optoelectronic plethysmography (OEP) system as a diagnostic instrument for quantifying cough peak flow is attributable to its proven accuracy and non-invasiveness [15]. The system’s design, devoid of mouthpieces, nose clips, face masks, or any interfacing components that might impede mouth and cheek movements, ensures a natural cough [5]. In the absence of this system, the evaluation of genuine alterations in chest wall kinematics or the immediate effects of the BS maneuver on CPF would be unfeasible.

The increase in CPF observed after applying the BS technique in the trunk inclination position (5.21 vs. 6.09 L/s; *p* < 0.05) indicates that the technique favours the generation of more effective intrathoracic pressures for air expulsion, even in healthy volunteers. This finding is consistent with recent studies that have shown an increase in CPF in populations with neuromuscular diseases and in the immediate postoperative period after interventions based on air stacking [16,17]. This effect can be explained by the increase in inspiratory capacity generated by the accumulation of air, which results in a mechanical advantage for the expiratory muscles during coughing.

With regard to lung volumes, the BS technique induced significant increases in chest wall tidal volume (V_T(CW)_) in both the supine and trunk inclination positions, with a more significant response in the inclination position (11.2%; *p* < 0.05). This finding is corroborated by other study, who demonstrated an increase in chest volume and upper alveolar recruitment in postures that favour diaphragmatic mobility and synergism between the respiratory muscles [18]. The greater effectiveness of BS in the inclination position may also be associated with greater thoracic compliance and reduced upper airway resistance in this posture [19].

Analysis of the respiratory compartments revealed that the POST_BS_ volume gain was concentrated in the rib cage compartment (CT), especially the P_RC_, with no significant change in abdominal volume. This pattern suggests that the technique predominantly induced an upper thoracic expansion, which may reflect the more favourable ventilatory mechanics of the prone position, associated with lower abdominal impedance and better chest wall movement [20]. In fact, optoelectronic studies have already shown that inclined postures promote a greater contribution from the thoracic compartment in total ventilation, especially during controlled insufflation techniques [21].

Analysis of the percentage contribution of volumes also showed a ventilatory redistribution in favour of the thoracic cavity in the inclination position, with a significant increase in the participation of the P_RC_ compartment and a proportional reduction in the abdominal contribution (*p* = 0.02). This redistribution is in line with the literature, which associates air stacking with an improved thoracic ventilatory pattern and increased collateral ventilation, especially when performed in optimized postures [22,23].

In addition, there was an increase in end-inspiratory volume (EIV) after the BS maneuver in both positions, although there were no statistical differences between them (Δ0.178 L vs. Δ0.145 L). This suggests that, regardless of posture, the BS technique is capable of promoting immediate volumetric gains, making it a viable resource for lung capacity training even in healthy populations. This effect was previously observed in recent clinical trials with patients on non-invasive ventilation, who showed similar increases in EIV after using BS as an adjunct technique [24].

Taken together, the findings reinforce the applicability of BS as a non-invasive and safe strategy to promote lung re-expansion, increased cough efficacy and favourable ventilatory redistribution, even in individuals without respiratory dysfunction. Its potentially increased effectiveness in the trunk inclination position should be considered in physiotherapy protocols aimed at lung re-expansion, including in outpatient settings.

## 5. Conclusions

In healthy subjects, the cough peak flow can be increased immediately after the application of the BS maneuver, as well as the total and operational chest wall volumes and breathing pattern.

Although the present study has contributed to addressing certain aspects related to the Breath Stacking maneuver, the extent to which this technique modulates pulmonary and thoracic wall compliance remains unclear. Further research is warranted to elucidate the underlying physiological mechanisms involved in the BS maneuver, particularly in both healthy individuals and populations with restrictive pulmonary disorders. Such investigations are essential to establish evidence-based applications and to optimize its therapeutic efficacy across clinical contexts.

## Figures and Tables

**Table 1:** Characteristics of each subject in relation to anthropometric data, absolute and predicted values in relation to lung function and respiratory muscle strength.

Subject	Sex	Age (Years)	BMI (kg/m^2^)	FVC (L)	FVC (%pred)	FEV_1_/FCV	FEV_1_/FCV (%pred)	MIP (cmH_2_O)	MIP (%pred)	MEP (cmH_2_O)	MEP (%pred)	SNIP (cmH_2_O)	SNIP (%pred)
1	M	23	25.72	4.91	92.98	0.83	85.67	128	93.49	142	96.81	88	75.12
2	M	21	22.22	4.68	80.36	0.84	84.66	132	95.30	138	93.06	89	75.43
3	M	27	24.97	4.81	84.44	0.86	85.23	130	97.23	130	90.63	95	82.27
4	M	27	25.84	5.60	111.73	0.85	85.23	132	98.72	130	90.63	87	75.35
5	M	20	24.58	4.78	88.80	0.85	86.09	140	100.50	140	93.89	102	86.14
6	M	21	21.56	4.65	90.84	0.83	86.94	142	102.52	153	103.17	96	81.36
7	M	24	23.89	3.89	97.86	0.84	84.61	95	96.30	102	101.03	89	99.30
8	F	23	23.81	3.46	81.25	0.84	83.93	110	110.96	110	108.29	90	100.17
9	F	27	23.93	3.58	84.70	0.83	83.15	98	100.85	115	116.00	95	106.78
10	F	25	20.43	4.34	98.70	0.82	83.05	100	101.88	120	119.58	87	97.31
11	F	22	22.95	3.68	93.77	0.83	85.20	95	95.36	90	88.07	96	106.59
12	F	22	23.65	3.98	99.19	0.83	84.92	102	102.38	98	95.90	88	97.71
13	F	24	19.56	4.10	105.49	0.81	84.89	97	98.33	96	95.08	97	108.23
14	F	27	23.23	4.23	105.58	0.82	83.85	115	118.34	100	100.87	92	103.41
Mean ± SD		23.79 ± 2.48	23.31 ± 1.84	4.26± 0.60	97.92 ± 7.42	0.83 ± 0.01	83.43 ± 1.34	115.4 ± 17.87	100.87 ± 6.64	118.86 ± 20.16	99.51 ± 9.49	92.21 ± 4.62	92.52 ± 12.65

BIM: Body mass index; FVC: forced vital capacity; L: litres; %pred: percentage of predicted; FEV_1_: forced expiratory flow on de first second; MIP: maximum inspiratory pressure; MEP: maximum expiratory pressure; SNIP: sniff nasal inspiratory pressure.

**Table 2:** Characteristics by sex of the subjects in relation to absolute and predicted values of lung function and respiratory muscle strength and anthropometric data.

Subjects	Male (7)	Female (7)	*p* ^§^
**Age (years)**	23.29 ± 2.87	24.29 ± 2.13	0.47
**BMI (kg/m^2^)**	24.11 ± 1.66	22.51 ± 1.76	0.10
**%FVC_pred_**	91.00 ± 12.06	95.53 ± 9.55	0.45
**%FEV_1_/FVC_pred_**	99.44 ± 1.75	98.74 ± 1.55	0.44
**%MIP_pred_**	97.73 ± 3.1	104.00 ± 7.93	0.07
**%MEP_pred_**	95.61 ± 4.94	103.40 ± 11.63	0.12

Data presented as mean and standard deviation. FVC: Forced Vital Capacity; FEV_1_: Forced expiratory volume in the 1st second; FEV_1_/FVC: Ratio of forced expiratory volume in the first second to forced vital capacity; MIP: Maximum inspiratory pressure; MEP: Maximum expiratory pressure; SNIP: Sniff nasal inspiratory pressure; m: metres; kg: kilograms; L: litres; %pred: Percentage of predicted; cmH_2_O: centimetres of water. ^§^ Intergroup differences.

## Data Availability

Data available on request from the authors. The data that support the findings of this study are available from the corresponding author (GAFG) upon reasonable request.

## Supplementary Materials

The following supporting information can be downloaded at: https://www.mdpi.com/article/10.3390/jfmk10040421/s1, Supplementary Material S1: Informed Consent Form.

## Abbreviations

The following abbreviations are used in this manuscript:

BS	Breath Stacking
CPF	Cough Peak Flow
FVC	Forced Vital Capacity
FEV_1_	Forced Expiratory Volume in 1 Second
MIP	Maximal Inspiratory Pressure
MEP	Maximal Expiratory Pressure
SNIP	Sniff Nasal Inspiratory Pressure
FRC	Functional Residual Capacity
OEP	Optoelectronic Plethysmography
V_T(CW)_	Tidal Volume of Chest Wall
ΔV_T(CW)_/Δt	Derived by the volume displaced by chest wall and time during the cough
QBi	Quiet Breathing initial
QB	Quiet Breathing
SVC	Slow Vital Capacity
PRE_BS_	Pre–Breath Stacking
POST_BS_	Post–Breath Stacking
BIM	Body Index Mass
V_T(rcp)_	Tidal Volume of Pulmonary Rib Cage
V_T(rca)_	Tidal Volume of Abdominal Rib Cage
V_T(Ab)_	Tidal Volume of Abdomen
P_RC_	Pulmonary Rib Cage
A_RC_	Abdominal Rib Cage
Ab	Abdomen
EIV	End-Inspiratory Volume
EEV	End-Expiratory Volume
