# Supplementary material for: Breath Stacking: Acute Effects on Cough Peak Flow and Chest Wall Volumes of Healthy Subjects
Source: J Funct Morphol Kinesiol. 2025 Oct 29;10(4):421. doi: 10.3390/jfmk10040421 (PMC12641795; doi:10.3390/jfmk10040421)
Supplement: Supplementary file 1 [file jfmk-10-00421-s001.zip › jfmk-3804116-supplementary.pdf]

FEDERAL UNIVERSITY OF RIO GRANDE DO NORTE
CENTER FOR HEALTH SCIENCES
DEPARTMENT OF PHYSIOTHERAPY

INFORMED CONSENT FORM

Clarifications

You are invited to participate in the research study titled: "Acute effects of the breath-stacking technique on lung volumes and cough peak flow in patients with Amyotrophic Lateral Sclerosis (ALS)", coordinated by researcher Ana Cristina de Medeiros Garcia Maciel.

Your participation is entirely voluntary, which means you may withdraw your consent at any time without any penalty or loss of benefits to which you are otherwise entitled.

This research is highly relevant for both healthy individuals and those diagnosed with ALS, as it aims to assess the acute respiratory effects of the Breath Stacking (BS) technique, which is designed to increase lung volumes in both sitting and supine positions. You will be part of the control group, composed of healthy individuals without neuromuscular disorders. Should you agree to participate, you will undergo the following procedures:

- An interview during which your age and health history will be recorded; your height and weight will be measured; and body fat will be assessed. Next, you will be asked to perform rapid breathing maneuvers to assess lung volumes and capacities. You will then be invited to sit on a backless bench with your arms resting on your legs and later lie down on an examination table. You will be filmed using six cameras, and reflective markers will be placed on your torso. These markers simply reflect the infrared light emitted by the cameras and do not cause any discomfort. One of the examiners will guide your positioning during the procedures. After each instruction, you will remain still for a few minutes while measurements are taken twice in each position. A cushioned mask will be placed on your face, and you will be asked to take three deep breaths. You will have rest periods between each repetition.

The risks associated with your participation are minimal, as strict eligibility criteria have been established. Possible discomforts may include fatigue during the tests, which will be minimized by interrupting or stopping the procedure if necessary and referring you to appropriate medical care.

The benefits of participating in this study include helping to identify how pulmonary volume-increasing techniques may support respiratory function in sitting and lying positions.

All collected information will remain confidential, and your name will not be disclosed at any time. The data will be stored securely, and any dissemination of results will ensure complete anonymity of participants.

Should you incur any expenses as a result of participating in the study, you will be reimbursed upon request.

In the event of proven harm resulting directly from your participation, you will have the right to receive appropriate compensation.

You will receive a copy of this consent form. Any questions about the study may be addressed to Ana Cristina de Medeiros Garcia Maciel, Department of Physiotherapy, Federal University of Rio Grande do Norte, Campus Universitário Lagoa Nova, P.O. Box 1524 – ZIP Code: 59072-970, Natal – RN – Brazil, or by phone: +55 (84) 3342-2027.

Questions regarding the ethical aspects of this study may be directed to the Research Ethics Committee, Praça do Campus Universitário, Lagoa Nova, P.O. Box 1666 – ZIP Code: 59072-970, or by phone: +55 (84) 3215-3135.

Statement of consent and signature

By signing this consent form, I acknowledge that I am not waiving any of my legal rights. I have read, or someone I trust has read to me, all the information provided in this form. I have had the opportunity to ask questions and have received satisfactory answers.

I understand that participation in this study is voluntary, and I have freely chosen to take part. My signature below indicates that the study and related procedures were clearly explained to me and that I understand and agree to participate.

I have received a signed and dated copy of this consent form, signed by both myself (or my legal representative) and the researcher. All pages have been initialed. I was also informed that a second original copy will be stored in the researcher's records.

Participant’s Name: ____

Participant’s Signature: ____

Date: ___ /___ /___

Legal Representative’s Name: ___________________________________

Legal Representation Type: (e.g., parent, guardian, legal proxy) ________

Participant’s Name: ____

Date: ___ /___ /___

I declare that I have properly and voluntarily obtained this participant’s (or legal representative’s) informed consent in accordance with Resolution CNS 466, December 12, 2012, items IV.3 to IV.6.

Name and Signature of the Responsible Researcher:

____

Date: ___ /___ /___
